# Differences in glycemic control between the treatment arms in cardiovascular outcome trials of type 2 diabetes medications do not explain cardiovascular benefits

**DOI:** 10.1186/s40545-020-00295-3

**Published:** 2021-04-15

**Authors:** Darren K. McGuire, Silvio E. Inzucchi, Odd Erik Johansen, Julio Rosenstock, Jyothis T. George, Nikolaus Marx

**Affiliations:** 1 University of Texas Southwestern Medical Center, Dallas, TX USA; 2 Section of Endocrinology, Yale University School of Medicine, New Haven, CT USA; 3 Clinical Development, Therapeutic Area Cardiometabolism, Boehringer Ingelheim, Hagaløkkveien 26, 1373 Asker, Norway; 4 Dallas Diabetes Research Center at Medical City and University of Texas, Southwestern Medical Center, Dallas, USA; 5 Clinical Development, Therapeutic Area Cardiometabolism, Boehringer Ingelheim, Ingelheim, Germany; 6 Department of Internal Medicine I, University Hospital Aachen, RWTH Aachen University, Aachen, Germany

**Keywords:** Type 2 diabetes, Cardiovascular outcome trials, Medication, Epidemiology, Risk reduction, SGLT-2 inhibitor, Bias

## Abstract

Hyperglycemia is an undisputed epidemiological risk factor for microvascular complications in both type 1 and type 2 diabetes, integral in their causal pathways. Importantly, interventions that reduce the hyperglycemic burden in patients with either type of diabetes reduce the risk of microvascular complications (e.g., retinopathy, nephropathy, neuropathy). Hence, for microvascular risk, hyperglycemia is a proven risk factor and a proven treatment target, as reflected by treatment recommendations and guidelines across most scientific societies world-wide. However, although reducing the hyperglycemic burden to reduce microvascular risk remains a cornerstone of care for patients with type 2 diabetes, this therapeutic imperative does not apply to cardiovascular risk mitigation. This latter aspect is important in the context of interpreting therapeutic impact of treating hyperglycemia on risk for macrovascular complications in patients with type 2 diabetes. This letter, in response to a previous paper, discuss how modest differential glucose control contribute little if anything to the results observed of contemporary cardiovascular outcome trials in type 2 diabetes.

## Main text

Hyperglycemia is an undisputed epidemiological risk factor for microvascular complications in both type 1 and type 2 diabetes, integral in their causal pathways [1, 2]. Importantly, interventions that reduce the hyperglycemic burden in patients with either type of diabetes reduce the risk of microvascular complications (e.g., retinopathy, nephropathy, neuropathy), as demonstrated by results from seminal trials such as the Diabetes Control and Complications Trial (DCCT) and United Kingdom Prospective Diabetes Study (UKPDS); respectively [3]. Hence, for microvascular risk, hyperglycemia is a proven risk factor and a clear treatment target, as reflected by treatment recommendations and guidelines across most scientific societies world-wide [2, 4].

In their recent manuscript *“Imbalance in glycemic control between the treatment and placebo groups in cardiovascular outcome trials in type 2 diabetes*” in the journal [5], Shimazawa and Ikeda inappropriately extrapolate the well-established epidemiological associations between hyperglycemia and cardiovascular outcomes in populations with type 2 diabetes to implications about therapeutic impact of treating hyperglycemia on risk for macrovascular complications in individual patients. Their views, with which we largely disagree, are exemplified by the statement “…the safety and efficacy of new hypoglycemic agents are potentially inflated because the participants in the placebo groups unexpectedly exhibited inferior glycemic control throughout the trial compared with the outcomes in the treatment groups.” We challenge their perspective on several fronts.

First, the authors’ position implies that glucose control (or lack thereof) is a surrogate for cardiovascular risk. For surrogacy to be valid, (a) the marker must be associated with outcomes; and (b), most importantly, changes in the marker must correlate with changes in outcomes. The first criterion is well established epidemiologically for cardiovascular risk in patients with type 2 diabetes: hyperglycemia consistently correlates with adverse cardiovascular outcomes [6]. However, the second criterion has failed to hold true, with results from a series of large, randomized clinical trials demonstrating no cardiovascular benefit (Action in Diabetes and Vascular Disease: Preterax and Diamicron Modified Release Controlled Evaluation [ADVANCE]; Veterans Affairs Diabetes Trial [VADT]) or even incremental risk (Action to Control Cardiovascular Risk in Diabetes [ACCORD]) to more versus less intensive glycemic control [7]. The totality of randomized trial evidence supports the conclusion that change in (or in this case, difference in) HbA1c is neither necessary nor sufficient to alter cardiovascular risk. This uncertainty underpinned the current and previous regulatory guidance in the US and in Europe to perform placebo-controlled trials (on top of standard of care) to evaluate cardiovascular safety and/or efficacy of new glucose lowering medications for type 2 diabetes [8].

Second, the authors’ contention that patients in the cardiovascular outcomes trials (CVOTs) had “inferior glycemic control” is neither supported by randomized trial data nor by contemporary American Diabetes Association/European Association for the Study of Diabetes guidelines since 2012 [9]. In accord with those recommendations, in older patients with long duration of diabetes and prevalent atherosclerotic cardiovascular disease and other comorbidities like kidney disease (such as those enrolled in these CVOTs of diabetes medications), more liberal HbA1c targets (< 8% or sometimes higher in selected high-risk groups) are advised. This now penetrant global practice may be reflected by the mean entry HbA1c for patients with type 2 diabetes with or at high risk for cardiovascular disease enrolled across the CVOTs of type 2 diabetes medications completed to date, and the observation that the achieved HbA1c in placebo groups for most of the completed trials averaged around 8%.

Finally, the authors claim a lack of statistical adjustments for differences in glycemic control in the reporting of results from these CVOTs. While this has not been done for all trials, it has been addressed in several. For example, in the EMPA-REG OUTCOME trial, assessing the CV safety and efficacy of the sodium-glucose cotransporter-2 (SGLT-2) inhibitor empagliflozin versus placebo, when added on top of standard of care, demonstrated a 38% relative risk reduction in cardiovascular mortality and a 35% relative risk reduction in risk for hospitalization for heart failure. Importantly, these effects did not differ from the overall trial findings when adjusted for HbA1c control both at baseline and by analysis of time-updated HbA1c achieved during the trial. So, the beneficial effects appeared largely independent of glucose control [10]. In addition, an exploratory mediation analysis of EMPA-REG OUTCOME data found that changes in hemoglobin/hematocrit and in albumin (indirect markers of plasma volume) were the most important mediators of the reduction in risk; any association between HbA1c and outcomes was quite modest [11].

The US and European regulatory guidance to perform placebo-controlled, on top of standard of care, cardiovascular safety trials, as opposed to head-to-head active comparator trials, invariably results in lower HbA1c in the active treatment groups. The contrast between groups in glycemic control is most evident at the beginning of the trial, with glucose control converging thereafter as patients in both arms are treated to the same glucose control targets with open label adjustments in antihyperglycemic medications. However, there is little evidence indicating that a relatively short period of differential glycemic control in individuals with relatively advanced cardiovascular disease will have a clinically relevant impact on their cardiovascular outcomes. However, we acknowledge that more intensive glucose control initiated early in the course of diabetes care may favorably affect long-term cardiovascular risk, as suggested by the results from the Epidemiology of Diabetes Interventions and Complications study (EDIC) and UKPDS legacy studies [12, 13].

## Conclusions

As primary investigators in several of the CVOTs of type 2 diabetes medications completed to date, we felt it important to provide some important counter opinions to those published by Shimazawa and Ikeda to inform a balanced interpretation of the robust cardiovascular data deriving from these recent trials. Although reducing the hyperglycemic burden to reduce microvascular risk remains a cornerstone of care for patients with type 2 diabetes, this therapeutic imperative does not apply to cardiovascular risk mitigation and such knowledge should inform interpretation of the CVOT results where differential glucose control most likely contributed little if anything to the results observed.

## Data Availability

Literature used to support the preparation of this commentary can be shared upon request.

## Abbreviations

DCCT: Diabetes Control and Complications Trial
UKPDS: United Kingdom Prospective Diabetes Study
ADVANCE: Action in Diabetes and Vascular Disease: Preterax and Diamicron Modified Release Controlled Evaluation
VADT: Veterans Affairs Diabetes Trial
ACCORD: Action to Control Cardiovascular Risk in Diabetes
CVOTs: Cardiovascular outcomes trials
SGLT-2: Sodium-glucose cotransporter-2
EDIC: Epidemiology of Diabetes Interventions and Complications Study
